# The association of hepatocyte growth factor (*HGF*) gene with primary angle closure glaucoma in the Nepalese population

**Published:** 2011-08-19

**Authors:** Mona S. Awadalla, Suman S. Thapa, Kathryn P. Burdon, Alex W. Hewitt, Jamie E. Craig

**Affiliations:** 1 Department of Ophthalmology, Flinders University, Flinders Medical Centre Adelaide, South Australia, Australia; 2 Nepal Glaucoma Eye Clinics, Tilganga Institute of Ophthalmology, Kathmandu, Nepal; 3 Centre for Eye Research Australia, University of Melbourne, Royal Victorian Eye and Ear Hospital, Melbourne, Australia

## Abstract

**Purpose:**

Genetic variation in the hepatocyte growth factor (*HGF*) gene has recently been associated with hyperopia, which is a known risk factor for primary angle closure glaucoma (PACG). This study aimed to investigate whether genetic variation in *HGF* is associated with primary angle closure glaucoma in the Nepalese population.

**Methods:**

One hundred six Nepalese patients with primary angle closure glaucoma and 204 matched controls were recruited. Twelve tag single nucleotide polymorphisms (SNPs) were selected and genotyped to cover the majority of common variation within *HGF*. Genotype and haplotype analyses were conducted in PLINK.

**Results:**

Four *HGF* SNPs were found to be significantly associated with PACG, rs5745718, rs12536657, rs12540393 and rs17427817 (p=0.002, 0.002, 0.0006, and 0.0006, respectively). In addition, haplotype analysis showed one common haplotype to be significantly associated with PACG (p=0.001) in this population.

**Conclusions:**

Genetic variation in *HGF* is associated with PACG in the Nepalese population. Additional replication studies in other populations are necessary to confirm this association and to further explore the role of *HGF* in the pathogenesis of this blinding disease.

## Introduction

Glaucoma represents a group of diseases with the common feature of slowly progressive destruction of the optic nerve with corresponding loss of the peripheral visual field [1]. Glaucoma is second only to cataract in causing blindness worldwide [2]. Importantly for patients with glaucoma, blindness is reported to be up to 25% higher in people with primary angle closure glaucoma (PACG), than open angle glaucoma worldwide [3].

PACG patients have been found to have particular anatomic biometric features including shallow anterior chambers [4], lens thickness and position [5], narrow iridiotrabecular drainage angles, short axial lengths [6], and hyperopic refractive error [7]. The most important risk factor is shallow anterior chamber depth [8], which has been found to correlate with older age, gender (commoner in females), and race (shallower in Eskimos and Asians than Caucasians and Africans) [9]. Asian populations are at higher risk of developing PACG than other groups [10], and the majority of bilaterally blind glaucoma patients live in China [11].

Amerasinghe et al. [12], found that siblings of Chinese patients with PACG have almost a 50% probability of having narrow angles. In another Chinese study, first degree relatives were also found to have 6–9 fold increased risk of developing ACG [13]. These studies suggest a genetic component to the risk of PACG. Several candidate genes have been studied in relation to PACG. The matrix-metalloproteinase-9 gene (*MMP9*), which is involved in scleral extra-cellular matrix (ECM) remodelling, was shown to be associated with PACG in Taiwanese patients [14], and in Australian patients [15], however several studies failed to replicate this association [16,17]. The Methylenetetrahydrofolate reductase (*MHTFR*) gene was also proposed to play a role in ECM remodelling of the anterior chamber in a PACG Pakistani cohort, secondary to C677T and A1298C polymorphisms [18,19]. The Membrane frizzled-related protein (*MFRP*) gene, which causes recessive nanophthalmos, has been studied in relation to PACG due to some similarities in the phenotypes, however no association was detected [20,21]. The inconsistencies in genetic findings between studies suggests that further evaluation of PACG genetics is warranted.

A recent study investigated the role of hepatocyte growth factor (*HGF*) in causing disruption of the emmetropization process within the eye [22]. The authors identified two single nucleotide polymorphisms (SNPs; rs12536657 and rs5745718) within *HGF* that were significantly associated with hyperopia. Since both angle closure glaucoma and hyperopia share the same feature of short axial length [23], we hypothesized that this gene may be involved in the development of PACG. The aim of our study was to investigate the association between tag SNPs of the *HGF* gene and primary angle closure glaucoma in the Nepalese population.

## Methods

Participants were recruited from the Nepal Glaucoma Eye Clinic, Tilganga Institute of Ophthalmology, Kathmandu, Nepal. Ethics approval was approved by the Institutional Review Committee of the Tilganga Institute of Ophthalmology (TIO), and is being conducted in accordance with the Declaration of Helsinki and its subsequent revisions. Informed consent was obtained from each individual. In total, 106 PACG cases, and 204 controls were recruited. Cases and controls were matched for sex and age although controls were slightly older than cases by design for this aging disease. All participants were from Nepal [24,25].

Each participant underwent a complete eye examination including; slit lamp examination of the anterior chamber, gonioscopy, best corrected visual acuity, measurement of intraocular pressure, fundus examination with special attention to optic disc parameters, and visual field assessment. Objective refraction was performed using a streak retinoscope (Beta 200, Heine, Germany), which was followed by a subjective refraction [24]. The diagnosis of PACG was based on the presence of glaucomatous optic neuropathy with cup:disc ratio ≥0.7, intraocular pressure more than 21 mmHg, peripheral visual loss, presence of at least 180 degrees of closed angle in which the trabecular meshwork is not visible on gonioscopy, which follow the International Society of Geographical and Epidemiological Ophthalmology (ISGEO) classification as described by Foster and colleagues [26].Controls were required to have none of the above characteristics, with no family history of glaucoma or previous glaucomatous operations. Participants with pseudophakia or secondary angle closure glaucoma caused by events such as uveitis, trauma or lens subluxation were excluded.

Genomic DNA was extracted from 2 ml of venous blood using the QiaAmp Blood Midi Kit (Qiagen, Valencia, CA). The two *HGF* SNPs (rs12536657, and rs5745718) identified by Veerappan et al. [22], as well as 10 other tag SNPs, were selected using the tagger program implemented in Haploview 4.2. SNPs were selected from the HapMap Han Chinese in Beijing, China (CHB) sample as the most closely related population available at the time of the study. Tag SNPs were chosen using pairwise tagging, to have an r^2^>0.8 with SNPs displaying a minor allele frequency of 5% in this population. SNPs previously reported to be associated with hyperopia were force included in the selection of tags. The 12 tag SNPs included: rs5745752 (A/G), rs5745718 (A/C), rs12536657 (A/G), rs2286194 (A/T), rs5745692 (C/G), rs12540393 (C/T), rs17427817 (C/G), rs12707453 (G/A), rs5745616 (A/G), rs3735520 (T/C), rs6942495 (G/C), and rs17501080 (C/A). A Bonferroni corrected p-value of 0.004 (0.05/12) was considered statistically significant.

Genotyping was conducted at the Australian Genome Research Facility, Brisbane, Australia, using the iPLEX Gold chemistry (Sequenom Inc., San Diego, CA) on an Autoflex mass spectrometer (Sequenom Inc.).

Differences in age and gender between cases and controls were assessed by *t*-test and χ^2^ test, respectively. All genetic analyses were conducted in PLINK [27]. SNPs were assessed for compliance with Hardy–Weinberg equilibrium using a χ^2^ test. Genetic association was assessed under allelic (allele 1 versus allele 2), dominant (1/1 genotype versus 1/2+2/2) and recessive (1/1 + 1/2 genotypes versus 2/2) models. Where fewer than 5 counts for a given genotype were observed, Fisher’s exact test was used, otherwise a χ^2^ test was used. Haplotypes across the two observed linkage disequilibrium blocks, as visualized in Haploview using the “solid spine” block definition, was also analyzed for association in PLINK.

## Results

Samples from 310 Nepalese individuals (comprising 106 cases and 204 matched controls) were genotyped. Demographic characteristics were similar between cases and controls (Table 1). Neither age nor gender differed significantly between cases and controls. The mean spherical equivalent for PACG cases was −0.15 diopters (D)±1.46, and in controls 0.09 D±0.31 (p=0.16). The ratio of females to males in both cases and controls was approximately 3:1.

**Table 1:** Demographic characteristics of the Nepalese cohort. SD=standard deviation

**Variables**	**Cases**	**Controls**	**p-value**
Number	106	204	-
Sex (% female)	76%	75%	0.85
Age in years: mean (SD)	57.3 (12.3)	60.3 (13.7)	0.07
Spherical equivalent in diopters: mean (SD)	−0.15 (1.46)	0.09 (0.31)	0.16
Intraocular pressure in mmHg: mean (SD)	21.36 (18)	12.8 (2.3)	>0.001
Cup: disc ratio; mean (SD)	0.8 (0.11)	0.2 (0.12)	>0.001

**Figure 1:**
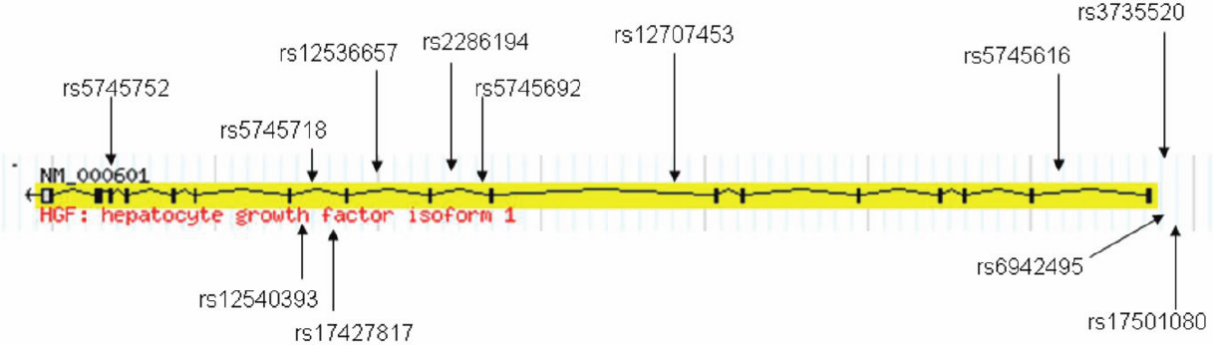
*HGF* gene idiogram depicting the location of all tagging SNPs, with the previously published hyperopia SNPs (above the line). Exons are indicated by gray or solid boxes and joined by introns indicated by lines. Figure adapted from the HapMap website.

All SNPs conformed to Hardy–Weinberg equilibrium in both cases and controls (p>0.05), and the call rate was >98%. The genotype counts and frequencies of all SNPs are shown in Table 2, along with the allelic association results. All 12 tag SNPs were located in the introns of *HGF*, with the physical location presented in Figure 1. Four SNPs reached statistically significant association with PACG; rs5745718 (p=0.002), rs12536657 (p=0.002), rs12540393 (p=0.0006), and rs17427817 (p=0.0006). After controlling for spherical equivalent via multivariate analyses, only three SNPs remained significantly associated with PACG; rs5745718 (p=0.003), rs12540393 (p=0.001), and rs17427817 (p=0.001; Table 2). Of the 12 SNPs, rs12540393 and rs17427817, were significantly associated under the dominant genetic model (p=0.001; Table 3). SNPs rs5745718 and rs12536657 also show nominal associations but did not survive correction for multiple testing.

**Table 2:** Genotype counts (n) and frequencies (%) of *HGF* SNPs in Nepalese samples and p-value for association under the allelic model, bold p-values are considered significant after bonferroni correction (p<0.004).

**SNP**	**Genotype**	**Case n (%)**	**Control n (%)**	**p-value**	Adjusted p-value*	**Odds ratio (95% CI)**
rs5745752	AA	16 (16.0)	30 (15.0)	0.754	0.694	0.9 (0.7–1.3)
	AG	44 (41.0)	90 (44.0)			
	GG	46 (43.0)	82 (41.0)			
rs5745718	AA	5 (4.0)	3 (1.0)	**0.002**	**0.003**	2.2 (1.3–3.5)
	AC	27 (26.0)	30 (15.0)			
	CC	71 (70.0)	163 (84.0)			
rs12536657	AA	5 (4.0)	3 (1.0)	**0.002**	0.009	2.1 (1.3–3.3)
	AG	29 (28.0)	34 (17.0)			
	GG	71 (68.0)	165 (82.0)			
rs2286194	AA	4 (4.0)	8 (4.0)	0.480	0.453	0.9 (0.6–1.3)
	AT	28 (26.0)	62 (31.0)			
	TT	74 (70.0)	132 (65.0)			
rs5745692	CC	0 (0.0)	1 (1.0)	0.139	0.998	0.2 (0.03–1.9)
	CG	1 (1.0)	6 (3.0)			
	GG	105 (99.0)	196 (96.0)			
rs12540393	CC	5 (5.0)	3 (3.0)	**0.0006**	**0.001**	2.2 (1.4–3.5)
	CT	34 (32.0)	37 (18.0)			
	TT	67 (63.0)	162 (79.0)			
rs17427817	CC	5 (5.0)	3 (2.0)	**0.0006**	**0.001**	2.2 (1.4–3.5)
	CG	34 (32.0)	37 (18.0)			
	GG	67 (63.0)	162 (80.0)			
rs12707453	GG	3 (3.0)	12 (6.0)	0.749	0.784	0.9 (0.6–1.4)
	GA	42 (40.0)	73 (36.0)			
	AA	60 (57.0)	117 (58.0)			
rs5745616	AA	16 (15.0)	21 (10.0)	0.540	0.469	1.1 (0.8–1.6)
	AG	43 (41.0)	91 (45.0)			
	GG	47 (44.0)	90 (45.0)			
rs3735520	TT	15 (14.0)	38 (19.0)	0.236	0.134	0.8 (0.6–1.1)
	TC	49 (47.0)	96 (47.0)			
	CC	41 (39.0)	68 (34.0)			
rs6942495	GG	22 (21.0)	51 (26.0)	0.745	0.878	0.9 (0.7–1.3)
	GC	56 (53.0)	95 (47.0)			
	CC	27 (26.0)	55 (27.0)			
rs17501080	CC	3 (3.0)	2 (1.0)	0.569	0.863	1.2 (0.7–2.0)
	CA	19 (18.0)	38 (19.0)			
	AA	83 (79.0)	162 (80.0)			

*Adjusted for spherical equivalent.

**Table 3:** Results of association tests for *HGF* SNPs in the case control analysis under dominant and recessive models with respect to the minor allele.

			**p-value**	
**SNPs**	**Minor allele**	**Nucleotide position**	**Dominant**	**Recessive**
rs5745752*	A	chr7: 81173396	0.635	0.954
rs5745718	A	chr7: 81185484	0.007	0.129
rs12536657	A	chr7: 81188144	0.006	0.127
rs2286194	A	chr7: 81193385	0.447	1
rs5745692	C	chr7: 81196202	0.271	1
rs12540393	C	chr7: 81202123	**0.001**	0.129
rs17427817	C	chr7: 81202371	**0.001**	0.129
rs12707453	G	chr7: 81207355	0.903	0.278
rs5745616*	A	chr7: 81236292	0.971	0.228
rs3735520*	T	chr7: 81238875	0.349	0.319
rs6942495*	G	chr7: 81240449	0.757	0.389
rs17501080	C	chr7: 81241632	0.881	0.342

95% CI=95% confidence interval. Bold p-values are considered significant after Bonferroni correction (p<0.004). *indicates χ^2^ test was used.

**Figure 2:**
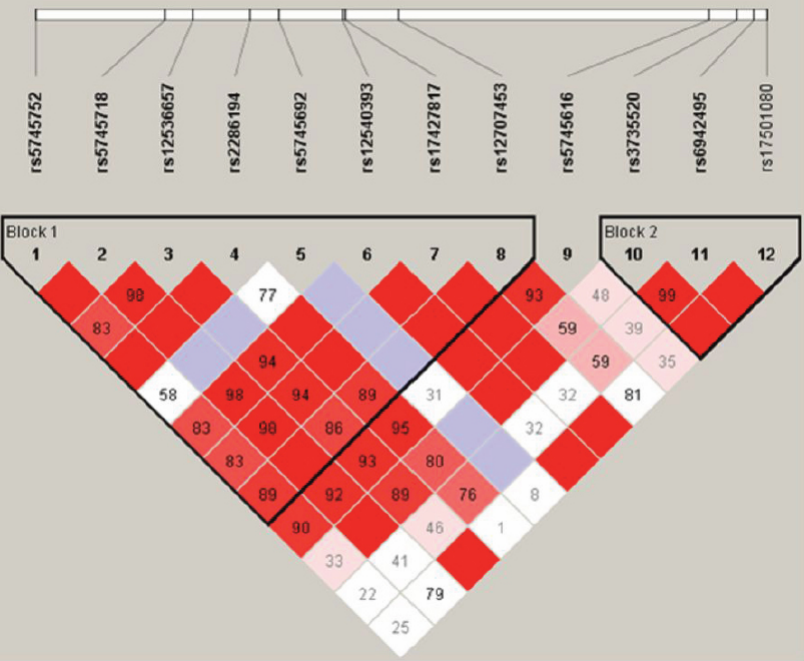
Linkage disequilibrium plot generated in Haploview shows the haplotype block structure using the solid spine definition. 100*׀D’׀ values are given. An empty cell indicated D’=1. The darker the red shading, the larger the ׀D’׀.

Haplotypic associations with PACG were also investigated. Two haplotype blocks were identified under the “solid spine” block definition, as displayed in Figure 2. Block 1 was defined by all SNPs between rs5745752 and rs5745616 (overall p-value=0.004), and block 2 as SNPs rs3735520 to rs17501080 (overall p-value of 0.024). In block 1 the frequency of the GAATGCCAG haplotype was significantly greater in cases than in controls (18.7% versus 9.5%, respectively, p=0.001) and remained significant after Bonferroni correction for the 6 haplotypes observed (p=0.006). Additionally, in block 2 the frequency of the CCA haplotype was found to be higher in cases than controls (15.2% versus 8.8%, respectively, p=0.017), but this difference was not statistically significant following Bonferronni correction (p=0.068; Table 4).

**Table 4:** Common haplotypes (>1% frequency) observed and association with PACG.

**Block**	**Haplotype**	**Case frequency**	**Control frequency**	**Odds ratio (CI 95%)**	**p-value**
1	A C G T G T G G A	0.22	0.23	0.9 (0.6–1.5)	0.877
	G A A T G C C A G	0.18	0.09	2.0 (1.3–3.3)	**0.001**
	G C G A G T G A G	0.18	0.19	0.9 (0.6–1.4)	0.594
	G C G T G T G A G	0.27	0.34	0.7 (0.5–1.0)	0.084
	A C G T G T G A G	0.01	0.03	0.3 (0.1–1.2)	0.074
	A C G T G T G A A	0.12	0.09	1.3 (0.7–2.1)	0.288
2	C G C	0.11	0.10	1.1 (0.6–1.9)	0.513
	C G A	0.35	0.38	0.9 (0.6–1.2)	0.424
	T C A	0.37	0.42	0.8 (0.6–1.2)	0.249
	C C A	0.15	0.08	1.8 (1.1–2.9)	**0.017**

An omnibus association test on overall p-value of 0.004 for block 1 and 0.024 for block 2. Bold values are considered to be statistically significant.

## Discussion

HGF protein has been found to play an important role in stimulating the growth and migration of various eye tissues including the corneal epithelium and endothelium, iris, retinal pigment epithelium, lens epithelium, and trabecular meshwork [28-31]. Hu and Ritch [32], revealed that the concentration of HGF in the aqueous humor was significantly higher in glaucomatous eyes than in cataract eyes, with no difference between open angle and angle closure glaucoma. Another study showed that *HGF* mRNA concentration was increased in rabbit lacrimal gland and corneal epithelium after injury of the cornea [33]. This supports the finding of Hu and Ritch [32] that increased HGF concentration in aqueous humor of glaucomatous eyes possibly reflects the functional effects of *HGF* on enhancement of aqueous flow and attempt to repair trabecular injury, rather than directly causing glaucoma.

The allelic association results displayed in Table 2 show four SNPs to be significantly associated with PACG (rs5745718, rs12536657, rs12540393, and rs17427817). The former two SNPs have recently been reported to be associated with hyperopia in an Australian Caucasian population [22]. Interestingly, the risk alleles for hyperopia, rs5745718(A) and rs12536657(A), in their study were the same as in our PACG study. This finding indicates a possible common pathway, or similarities between hyperopia and PACG which are known to share similar biometric features including short axial length [22,34]. However, all four SNPs in our PACG study were significantly associated with PACG independent of spherical equivalent, so the association appears to occur through a mechanism beyond an indirect association with hyperopia in this population.

The GAATGCCAG haplotype of block 1 showed significant association with PACG (p=0.001). This haplotype contains the associated risk allele of each of the significantly associated SNPs; rs5745718(A), rs12536657(A), rs12540393(C), and rs17427817(C). The frequency of this haplotype was also found to be higher in the PACG patients (18%) than in healthy controls (9%), reflecting the allele frequencies of these four SNPs. These alleles do not occur on any other common haplotype in this population. The association of the haplotype in block 2 is likely due to the linkage disequilibrium between the associated SNPs in block 1 and SNPs in block 2.

The role of *HGF* in PACG remains unknown. Further work is needed to determine its involvement in the pathogenesis of this blinding disease. The similar findings in hyperopia indicate that it may be involved in influencing the structure of the eye and thus predisposing those with short axial length to the risk of angle closure. It is unlikely that the tag SNPs assayed here are the functional variants. All four tag SNPs are located in introns of *HGF* and are likely to be in linkage disequilibrium with actual functional variants. The causative variant will likely be found on the background of the GAATGCCAG haplotype.

The main limitation of this study is the use of CHB to tag the Nepalese samples, however we found that the allele frequencies of the SNPs were remarkably similar between the two groups and thus they are likely to be quite similar.

To our knowledge, this is the first report to identify an association between *HGF* and PACG. Additionally the underlying genetic etiology of PACG in people of Nepalese descent has not been previously studied. In conclusion, this study revealed an association between the *HGF* gene and PACG. Future replication studies in different populations are necessary to confirm this association and to further explore the role of *HGF* in the pathogenesis of the disease.
